# Unforeseen misuses of bed nets in fishing villages along Lake Victoria

**DOI:** 10.1186/1475-2875-7-165

**Published:** 2008-08-27

**Authors:** Noboru Minakawa, Gabriel O Dida, Gorge O Sonye, Kyoko Futami, Satoshi Kaneko

**Affiliations:** 1 Institute of Tropical Medicine, Nagasaki University, Nagasaki, Japan; 2 School of Public Health, Maseno University, Maseno, Kenya; 3 International Centre for Insect Physiology and Ecology, Mbita, Kenya

## Abstract

**Background:**

To combat malaria, the Kenya Ministry of Health and nongovernmental organizations (NGOs) have distributed insecticide-treated nets (ITNs) for use over beds, with coverage for children under five years of age increasing rapidly. Nevertheless, residents of fishing villages have started to use these bed nets for drying fish and fishing in Lake Victoria. This study investigated the extent of bed net misuse in fishing villages.

**Methods:**

Seven fishing villages along the lake were surveyed to estimate how widely bed nets were being used for fishing and drying fish. Villagers were asked why they used the bed nets for such purposes.

**Results:**

In total, 283 bed nets were being used for drying fish. Of these, 239 were long-lasting insecticidal bed nets (LLIN) and 44 were non-long-lasting insecticidal bed nets (NLLIN). Further, 72 of the 283 bed nets were also being used for fishing. The most popular reasons were because the bed nets were inexpensive or free and because fish dried faster on the nets. LLINs were preferred to NLLINs for fishing and drying fish.

**Conclusion:**

There is considerable misuse of bed nets for drying fish and fishing. Many villagers are not yet fully convinced of the effectiveness of LLINs for malaria prevention. Such misuses may hamper the efforts of NGOs and governmental health organizations.

## Background

The World Health Organization (WHO) announced the Roll Back Malaria (RBM) movement in 1998, with the goal of decreasing malaria deaths by half by 2010 [1]. Several field trials demonstrated that insecticide-treated nets (ITNs) are effective in reducing malaria-related mortality in sub-Saharan Africa [2]; thus, ITNs have become a major tool in RBM. In Kenya, ITNs have been mainly distributed to pregnant women and children under five years of age, either free of charge or at subsidized prices, through programmes of the Kenya Ministry of Health and nongovernmental organizations (NGOs) [3,4]. Consequently, ITN coverage for children under five years of age has increased rapidly from 7% in 2004 to 67% in 2006; this increase has been associated with a 44% reduction in malaria deaths [5].

**Figure 1:**
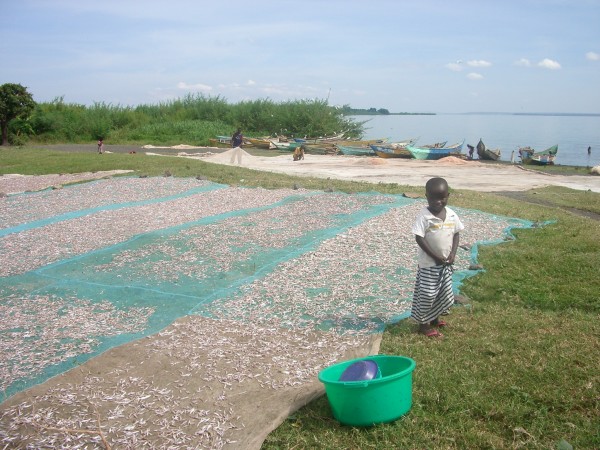
Omena fish spread on bed nets on a beach by Lake Victoria.

Nevertheless, a study in western Kenya found that 30% of bed net recipients did not adhere to net use [6,7]. Net use tends to decrease during hot weather. Further, ITNs are sometimes used for other purposes such as wedding dresses or fishing in Zambia [8]. Bed nets have also been observed being used for drying a small zooplanktivorous Dcyprinid (*Rastrineobola argentea*, called "omena" in the local language) in fishing villages in the Kenyan part of the Lake Victoria basin (Figure 1), where malaria is endemic. Traditionally, these fish have been dried on papyrus sheets. However, the extent of bed net misuse for this purpose is unknown. The widespread misuse of the nets might hinder the RBM goal. Thus, this study investigated how widely bed nets were used for fishing and drying fish in villages along Lake Victoria in western Kenya.

## Methods

### Study area

The study was conducted in seven major fishing villages in the Gambe West sub-district of Suba District, western Kenya. The area is approximately 76.6 km^2^. Most residents in the sub-district depend on fishing and traditional small-scale farming. The primary targets of the local fishery are Nile perch (*Lates niloticus*), Nile tilapia (*Orechromis niloticus*), and omena. Omena and Nile perch each accounted for 43% of the catch in Lake Victoria during the period of 1980 to 2005 [9]. Although Nile perch accounted for > 90% in volume of Kenya's total fish exports during the period of 1985 to 2005 [9], omena is an important protein source for locals [10].

Two rainy seasons occur annually from approximately March to June and October to November, but the periods vary by year. Malaria is the leading cause of morbidity and mortality of children in the region [11]. Three species of vectors are known: *Anopheles arabiensis*, *Anopheles gambiae *and *Anopheles funestus *[12].

### Bed net survey

Each village had its own fish-landing beach, and nearly all captured omena fish were spread out and dried on the beach. The beaches were visited three times in early morning during "young" moon periods in February and March 2008 (during the rainy season). Omena fishing is not active during full moon periods. Fishermen use lamps to attract omena towards the boats during the night; this method is not effective under a full moon.

When a sheet for drying fish was found, its material was categorized as papyrus, fishing net, bed net, or other. Bed nets were further categorized as long-lasting insecticidal bed net (LLIN) or non-long-lasting insecticidal bed net (NLLIN). NLLINs include nets with and without periodical insecticidal treatments. The size of each sheet in square metres was measured using a tape measure with the permission of the owner. When a sheet had been created from multiple bet nets or fishing nets, the number of nets was counted. Bed nets and fishing nets were measured separately when a sheet consisted of both materials.

To determine whether villagers preferred LLINs or NLLNs for drying fish, information on the availability of both types of bed net in the area was necessary. This background information was obtained from a previous survey that was designed to estimate bed net coverage in the study area in August 2007 (Dida, unpublished data). In total, 111 houses were visited, and the numbers of LLINs and NLLNs were counted. The sources of bed nets (i.e., stores, NGOs, or health facilities) and the number of residents in each house were also recorded.

Owners of fish-drying sheets that consisted of a bed net were asked the following questions: where and when the bed net was acquired, whether any bed nets were currently used in the house, why bed nets were used for drying fish, whether bed nets had ever been used for fishing in the lake, and when they started using the bed nets for drying fish. The investigated sheets were numbered to avoid duplicating data collection during a subsequent visit.

In June 2006, an NGO distributed LLINs mainly to children in the villages. This NGO provided information on the number of LLINs distributed to each village. The interviewees were also asked whether the NGO provided the LLINs in use on the beaches.

### Statistical analysis

The total sizes of bed nets, fishing nets, and papyrus sheets were estimated for each beach. The mean total sizes were compared using repeated-measures analysis of variance (ANOVA). The Tukey-Kramer honestly significant difference (HSD) test was used for post-hoc multiple comparisons.

A paired t-test was used to test the difference in the number of LLINs and NLLINs used for drying fish. The values were log-transformed because of heteroscedasticity. The proportion of LLINs to NLLNs was calculated for each beach and also obtained for houses. These values were arcsine-transformed and then compared between beaches and houses using a chi-square test to determine whether either type of bed net was used preferentially for drying fish.

The difference in number between the types of bed nets used for fishing was compared using a Wilcoxon test because transformation did not stabilize the variances. To determine whether either type of bed net was preferably used for fishing, the proportion of LLINs to NLLNs used for fishing was compared with that for drying fish using a paired test.

A paired t-test was also used to compare the number of bed nets obtained from NGOs and health facilities with that of bed nets acquired from stores. The proportion of nets from NGOs or health facilities to those from stores was compared between the beaches and the houses using a chi-square test. The significance level was 0.05 for all tests.

## Results

The total number of sheets used for drying fish was 166 at the seven fishing beaches, and the total area was 8295.8 m^2 ^(Table 1). Nearly half of the sheet area was either bed nets or fishing nets; papyrus sheets made up only 9.5% of the sheet area. Bed nets accounted for 15.0 to 83.8% of the total sheet area among the beaches. The repeated-measures ANOVA indicated that the mean sheet area varied significantly among the materials (F = 4.55; df = 2, 20; P = 0.034). The post-hoc multiple comparisons indicated that the area of fishing nets was significantly greater than that of papyrus sheets, but the differences between bed nets and the other materials were insignificant.

**Table 1:** Total and mean areas (square metres per village) of bed nets, fishing nets, and papyrus sheets used for drying fish (n = 7).

Material	Area	%	Mean (SE)
Bed nets	3686.8	44.4	515.8 (82.7)
Fishing nets	3770.8	45.5	550.5 (116.5)
Papyrus sheets	788.1	9.5	112.6 (75.1)
Other	50	0.6	7.1 (4.9)
Total area	8295.8	100.0	-

Of 166 sheets, 87 consisted of 283 bed nets, of which 238 were LLINs and 44 were NLLINs (Table 2). The paired t-test revealed that significantly more LLINs were found on the beaches compared with NLLINs (t = 7.11, df = 12, P < 0.001). In total, 220 bed nets were found in 111 houses. Of these, 145 bed nets were LLINs, and 75 were NLLINs (Table 3). The proportion of LLINs to NLLINs used for drying fish was significantly greater than that of LLINs to NLLINs in the houses (chi-square test: χ^2 ^= 23.50, P < 0.001). Of 220 bed nets, 87 were obtained from stores and 130 were from NGOs or health facilities (the information on three nets was not available). The mean numbers of total residents and children under five years of age were 3.6 and 0.6 per house, respectively.

**Table 2:** Numbers, percentages, and means (per village) of bed nets used for drying fish and fishing (n = 7) and their sources.

	Number	%	Mean (SE)
Bed nets used for drying fish			
LLIN	239	84.5	34.1 (8.2)
NLLIN	44	15.5	6.3 (1.8)
Total	283	100.0	-
Bed nets used for fishing			
LLIN	68	94.4	9.7 (2.4)
NLLIN	4	5.6	0.6 (0.3)
Total	72	100.0	-
Source of bed nets			
NGOs or health facilities	239	84.5	34.1 (7.8)
Stores	44	15.5	6.3 (2.2)
Total	283	100.0	-
Year when bed nets were acquired			
2008	2	0.7	-
2007	190	67.1	-
2006	74	26.1	-
2003, 2004 and 2005	17	6.0	-
Total	283	100.0	-

**Table 3:** Numbers, percentages, and means (per house) of residents and bed nets in houses (n = 111) and their sources.

	Number	%	Mean (SE)
Bed nets in houses			
LLIN	145	65.9	1.3 (0.1)
NLLIN	75	34.1	0.7 (0.1)
Total	220	100.0	2.0 (0.1)
Source of bed nets			
NGOs or health facilities	87	40.1	-
Stores	130	59.9	-
Total	217*	100.0	
Residents in houses			
Children under 5 years of age	70	17.4	0.6 (0.1)
Persons above 5 years of age	334	82.6	3.0 (0.1)
Total	404	100.0	3.6 (0.2)

*Information on three bed nets was not available.

Among the bed nets found on the beaches, 72 (24.5% of the total nets) nets had been used for fishing. Of these, 68 were LLINs and four were NLLINs. Significantly more LLINs than NLLINs were used for fishing (Wilcoxon test: χ^2 ^= 6.52, P = 0.013). The proportion of LLINs that were used for fishing was significantly greater than that of LLINs that were used for drying fish (t = 2.76, df = 12, P = 0.04).

In total, 239 (84.5%) bed nets were obtained either free of charge or at subsidized prices from NGOs and local health facilities, and only 44 (15.5%) nets were purchased at stores. The mean number of bed nets obtained from NGOs or health facilities was significantly greater than that of the bed nets obtained from stores (t = 8.84, df = 12, P < 0.001). The proportion of nets obtained from NGOs or health facilities to those obtained from stores was significantly greater for the beaches than for the houses (chi-square test: χ^2 ^= 38.33, P < 0.001). Of 283 bed nets found on the beaches, 74 (26.1%) and 190 (67.1%) nets were acquired in 2006 and 2007, respectively.

All 87 owners of bed nets found on the beaches were interviewed as to why they used the bed nets for drying fish. Of these, 85 owners answered the interview questions. The most popular reasons were because fish dried faster and bed nets were cheap or free (Table 4). Of the 85 owners, only seven were not using bed nets in their houses. Most owners started using bed nets for drying fish in 2006 and 2007.

**Table 4:** Reasons for using bed nets for drying fish and the year that this practice was started.

	Number	%
Reasons for using bed nets for drying fish		
Fish dry faster on bed nets	64	75.3
Inexpensive	38	44.7
Fish do not stick to bed nets	25	29.4
Fish dry straight on bed nets	17	20.0
No other materials for drying fish	16	18.8
Easy to obtain from NGOs	15	17.6
Have enough bed nets	14	16.5
Original colour of fish is retained on bed nets	8	9.4
Strong	6	7.1
Other	7	8.2
Number of interviewees	85	-
Year when started to use bed nets for fish drying		
2008	3	3.7
2007	32	39.0
2006	43	52.4
2005	4	4.9
Number of interviewees	82	100.0

The single NGO distributed 1040 LLINs in six villages (the number of bed nets distributed in one village was not available), of which 170 (16.3%) were being used for drying fish. Among the villages, the percentages of bed net used for drying fish ranged from 5.9 to 43.3%. Of 239 LLINs found on the beaches, 71.1% were from that particular NGO.

## Discussion

A considerably large number of bed nets were used for drying fish and fishing in the study area adjacent to Lake Victoria. Although the misuse of bed nets for fishing has been reported from Zambia without details [8], their use for drying fish was previously unknown. The traditional method of using papyrus sheets for drying fish was no longer popular in the study area and had been replaced with the method using bed nets and fishing nets.

The interviews with bed net owners suggested that bed nets have clear advantages over papyrus sheets for drying fish. Whereas the price of a papyrus sheet ranged between 150 and 200 Kenya shillings (Ksh), a bed net could be obtained from an NGO free of charge or from local health facilities at subsidized prices (usually 50 Ksh). Bed nets were readily available from these organizations, but papyrus sheets were only available in the weekly market in the major local town. In fact, nearly 85% of the bed nets found on the beaches were from NGOs and local health facilities. The villagers also indicated that fish dried faster on the bed nets, which provided greater aeration when laid on grass than did papyrus sheets. They also noted that the fish dried straighter on bed nets, which increased the commercial value of the fish.

A larger proportion of LLINs than NLLINs was used for drying fish than shown by the background information from the houses. This suggests that villagers preferred LLINs because the materials used for LLINs are stronger than those used for NLLINs and are more suitable for use outdoors. Moreover, approximately one-fourth of the bed nets found on the beaches were also being used for fishing in the lake, and the proportion of LLINs used for fishing was greater than that for drying fish. Only four NLLINs were being used for fishing. This is reasonable because fishing requires stronger materials than does fish drying. Although LLINs are weaker than real fishing nets, they are much cheaper or free and are at least strong enough to catch small fish such as omena. After fishing, the nets can be used for drying fish while the nets are also being dried on the beaches. For villagers who buy nets at subsidized prices, the use of LLINs as a disposable fishing net must be cost effective, although LLINs used for fishing purposes wear out much faster than those used inside the home. Consequently, as NGOs and health facilities distribute more LLINs, more LLINs may be used for fishing and fish drying.

Over 70% of LLINs found on the beaches were from the single NGO; > 15% of the nets distributed by that NGO were used on the beaches, even though the nets were mainly provided to children. The interviews clearly indicate that misuse of the nets started in the period when the Kenya Ministry of Health and NGOs began distributing LLINs. Although data were unavailable, it seems that LLINs were not popular in this area before they began distributing the nets.

The proportion of bed nets obtained from NGOs or health facilities to those from stores was greater for the beaches than for the houses. This suggests that villagers preferentially use free or inexpensive bed nets for fishing purposes because the practice does not cost them. For some villagers, fishing might be more important than protection from mosquitoes. Alternately, villagers concerned for their health might have bought bed nets for house use before the NGO's distribution; therefore, the nets provided by the NGO could have been extra nets.

Nearly 15% of the interviewees answered that they used bed nets on the beaches because the nets were extra, and > 80% reported that they had bed nets in their houses. However, it is difficult from this survey to conclude whether there were enough bed nets to cover all residents in the houses because information on the number of residents and bed nets in individual houses was not available. However, a previous survey of houses found means of 2.0 bed nets and 3.6 residents per house. Although the house survey was conducted in 2007 (six to seven months before the beach survey), the villagers had started to use most of the bed nets found on the beaches in 2006 and 2007. This suggests that there were not enough bed nets to cover all residents when they started to use the nets for fishing.

Bed nets may be reused on beaches after being used in houses considerably. However, the results from this study deny this possibility. Over 90% of bed nets found on the beaches were not older than two years approximately, and nearly 70% them were likely newer than one year old. LLINs were designed to last more than two years. Considering these and the timing that misuse of the nets started in the period when the Kenya Ministry of Health and NGOs began distributing LLINs, it is suspected that the bed nets had been little used in houses. Worn-out bed nets with holes are not suitable for fishing, at least.

Because omena fishing is important in villages along the lake [10], misuses of bed nets must be common throughout the lake region. Breeding habitat for malaria vectors is closely associated with lakeshores [12,13], and malaria transmission is high near the lake. The misuses of bed nets must be a substantial drawback for malaria-control programmes involving LLINs in the region.

## Conclusion

The misuse of bed nets for drying fish and fishing is considerable in the study area. Many villagers are not yet fully convinced of the effectiveness of LLINs for malaria prevention. Misuses of bed nets may hamper the efforts of NGOs and governmental health organizations for malaria prevention.

## Competing interests

The authors declare that they have no competing interests.

## Authors' contributions

NM initiated the study and drafted the manuscript. GD and GS initially identified the misuses of bed nets on the beaches and led the field survey. KF and SK organized and analysed the data. All authors have read and approved the final manuscript.

## References

[B1] Nabarro DN, Tayler EM (1998). The "roll back malaria" campaign. Science.

[B2] Lengeler C (2004). Insecticide-treated bed nets and curtains for preventing malaria. Cochrane database of systematic reviews (Online).

[B3] Noor AM, Amin AA, Akhwale WS, Snow RW (2007). Increasing coverage and decreasing inequity in insecticide-treated bed net use among rural Kenyan children. PLoS Med.

[B4] Wacira DG, Hill J, McCall PJ, Kroeger A (2007). Delivery of insecticide-treated net services through employer and community-based approaches in Kenya. Trop Med Int Health.

[B5] Fegan GW, Noor AM, Akhwale WS, Cousens S, Snow RW (2007). Effect of expanded insecticide-treated bednet coverage on child survival in rural Kenya: a longitudinal study. Lancet.

[B6] Alaii JA, Borne HW van den, Kachur SP, Shelley K, Mwenesi H, Vulule JM, Hawley WA, Nahlen BL, Phillips-Howard PA (2003). Community reactions to the introduction of permethrin-treated bed nets for malaria control during a randomized controlled trial in western Kenya. Am J Trop Med Hyg.

[B7] Alaii JA, Hawley WA, Kolczak MS, ter Kuile FO, Gimnig JE, Vulule JM, Odhacha A, Oloo AJ, Nahlen BL, Phillips-Howard PA (2003). Factors affecting use of permethrin-treated bed nets during a randomized controlled trial in western Kenya. Am J Trop Med Hyg.

[B8] Hopkin M (2008). The big push. Nature.

[B9] Okechi JK, Owili M (2006). An overview of fisheries and aquaculture in Kenya.

[B10] Geheb K, Binns T (1997). "Fishing farmers" or "farming fishermen"? The quest for household income and nutritional security on the Kenyan shores of Lake Victoria. African Affairs.

[B11] Mutero CM, Ouma JH, Agak BK, Wanderi JA, Copeland RS (1998). Malaria prevalence and use of self-protection measures against mosquitoes in Suba District, Kenya. East Afr Med J.

[B12] Minakawa N, Seda P, Yan G (2002). Influence of host and larval habitat distribution on the abundance of African malaria vectors in western Kenya. Am J Trop Med Hyg.

[B13] Minakawa N, Sonye G, Dida OG, Futami K, Kaneko S (2008). Recent reduction in the water level of Lake Victoria has created more habitats for *Anopheles funestus*. Malar J.

